# A MEMS-based Benzene Gas Sensor with a Self-heating WO_3_ Sensing Layer

**DOI:** 10.3390/s90402895

**Published:** 2009-04-21

**Authors:** Ming-Tsun Ke, Mu-Tsun Lee, Chia-Yen Lee, Lung-Ming Fu

**Affiliations:** 1 Department of Energy and Refrigerating Air-conditioning Engineering, National Taipei University of Technology, Taiwan, Taiwan 106; E-Mails: mtke@ntut.edu.tw (M.-T.K.); me586032037@gmail.com (M.-T.L.); 2 Department of Materials Engineering, National Pingtung University of Science and Technology, Pingtung, Taiwan 912

**Keywords:** benzene, gas sensor, MEMS, micro-heater, WO_3_ thin film

## Abstract

In the study, a MEMS-based benzene gas sensor is presented, consisting of a quartz substrate, a thin-film WO_3_ sensing layer, an integrated Pt micro-heater, and Pt interdigitated electrodes (IDEs). When benzene is present in the atmosphere, oxidation occurs on the heated WO_3_ sensing layer. This causes a change in the electrical conductivity of the WO_3_ film, and hence changes the resistance between the IDEs. The benzene concentration is then computed from the change in the measured resistance. A specific orientation of the WO_3_ layer is obtained by optimizing the sputtering process parameters. It is found that the sensitivity of the gas sensor is optimized at a working temperature of 300 °C. At the optimal working temperature, the experimental results show that the sensor has a high degree of sensitivity (1.0 KΩ ppm^−1^), a low detection limit (0.2 ppm) and a rapid response time (35 s).

## 1. Introduction

The presence of volatile organic compounds (VOCs) in indoor environments can cause irritation of the ears, nose and throat and may produce an unpleasant odor [1]. Of these VOCs, benzene (C_6_H_6_) arouses particular concern due to its toxicity and carcinogenic properties. Benzene is carcinogenic via all routes of exposure and is known to be a major cause of leukemia and lymphomas [2].

VOCs are generally detected using some form of conventional gas chromatography (GC) technique combined with mass spectrometry (MS). However, such methods are unable to provide benzene exposure information on a real-time basis. As a result, a number of researchers have developed optical sensors for gas quantification applications [3–6]. However, the optical arrangements of these sensors tend to be somewhat bulky and elaborate. Over the past decade, MEMS technologies and micromachining techniques have facilitated the miniaturization of all manner of devices, including gas sensors. The advances made in MEMS technology now enable the acquisition of sensing information at a micro-scale level [7–12].

Many researchers have demonstrated the suitability of thin or thick WO_3_ films as sensing layers for gas monitoring applications [13–18]. Wang *et al.* [13] fabricated monolithic thin-film metal-oxide gas sensor arrays to detect the presence of VOCs. In their study, microfabrication techniques and a reactive radio frequency (RF) sputtering process were used to deposit SnO_2_, ZnO and WO_3_ sensing layers on silicon substrates to monitor the concentrations of benzene, toluene and methanol, respectively, in air. Tomchenko *et al.* [14,15] fabricated WO_3_ thick-film gas sensors using a screen-printing technique. The results showed that a sensing layer with a thickness of 15 μm optimized the sensing performance over a concentration range of 2 to 300 ppm NO gas at a working temperature of 300 °C. Penza *et al.* [16] also presented a NO_x_ gas sensor featuring a WO_3_ sputtered thin film. In this case, it was shown that the sensitivity of the device was optimized by specifying the thickness of the WO_3_ sensing layer as 4800 Å and the working temperature as 250 °C [17]. Lee *et al.* [18] presented a WO_3_-based nanocrystalline thick film gas sensor. The results showed that the nano-scale grain size of the WO_3_ sensing layer enhanced the sensitivity of the device, resulting in a good sensitivity to low level NO_x_ concentrations (0.5 – 30 ppm) at a working temperature of 350 °C.

WO_3_ possesses good sensing characteristics for the detection of NH_3_ and O_3_ in air [19–21]. Llobet *et al.* [19] used a drop-coating method to fabricate WO_3_ sensors to monitor NH_3_ in air. The sensitivity of the device was found to be enhanced at a working temperature of 300 °C. O_3_ gas sensors have been successfully developed by printing WO_3_ porous layers directly onto impermeable substrates [20] or by using a RF reactive magnetron sputtering technique [21].

This study employs a straightforward MEMS-based fabrication process to realize a benzene sensor featuring a quartz substrate, an integrated Pt micro-heater, Pt interdigitated electrodes (IDEs) and a sputtered WO_3_ layer with a micron-scale grain size,. It is found that the sensitivity of the gas sensor is optimized at a working temperature of 300 °C. At the optimal working temperature, the experimental results show that the sensor has a high degree of sensitivity (1.0 KΩ ppm^−1^), a low detection limit (0.2 ppm) and a rapid response time (35 s).

## 2. Sensor Design

### 2.1. Micro-heater

The sensor developed in this study operates on the principle that changes in the coverage of the adsorbed or chemisorbed gas species on the sensing film cause a detectable change in the electrical conductance of the film. Gas sensing devices are typically designed to operate at elevated temperatures in order to activate the reactions which produce the sensor response and to reduce humidity effects [22]. However, a drawback of such devices is that the observed response may actually be the result of the presence of more than one gas in the atmosphere. Therefore, to enhance the “selectivity” of a device, its operating temperature must be optimized in order to reduce the effects of competing reactions. In the present study, a Pt micro-heater is fabricated on the sensing layer [23]. When an electrical voltage is applied to this heater, its temperature increases, generating a simultaneous heating effect in the sensing layer immediately above it. The optimal operating temperature for the sensor is determined by varying the temperature of the heater, i.e. by varying the applied voltage, and comparing the detection sensitivities at different temperatures.

### 2.2. Design

**Figure 1.**
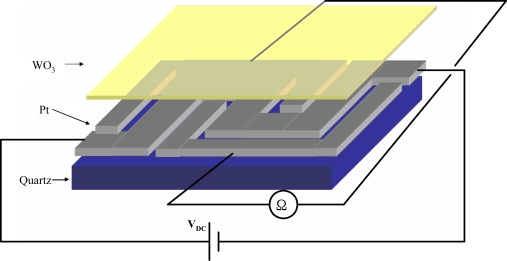
Schematic illustration of benzene sensor with integrated micro-heater and IDEs (Sensor size: 1.0 mm × 1.0 mm × 100 μm).

As shown in Figure 1, the benzene sensor developed in this study comprises a quartz substrate, Pt micro-heater/ IDEs and a WO_3_ sensing layer. The micro-heater provides an instantaneous and precise temperature control capability, while the IDEs facilitate the direct electrical measurement of the change in conductivity of the sensing layer induced by the presence of benzene in the atmosphere. The grain size of the sputtered WO_3_ sensing layer is very small, and therefore both the sensitivity and the response time of the device are enhanced due to the increased contact area between the catalyst grains and the sensed gas.

## 3. Fabrication

### 3.1. Thin film deposition

The WO_3_ films were prepared using an RF magnetron sputtering system with a W target of 99.99% purity [24–26]. The oxide layer was sputtered on a quartz substrate located at a distance of 11.4 cm from the W target. Sputtering was performed under a gas pressure of 0.01 torr with the target maintained at a constant RF power of 200 W. The reactive sputtering gas was a mixture of argon (50%) and pure oxygen (50%). Prior to deposition, the chamber was pumped to a background pressure of 10^−6^ torr for 1 h and a pre-sputtering process was performed for 10 min to remove any traces of contamination from the target surface. Subsequently, RF sputtering was performed for 10 h, resulting in a WO_3_ film with a thickness of approximately 4.4 μm.

### 3.2. Microfabrication

**Figure 2.**
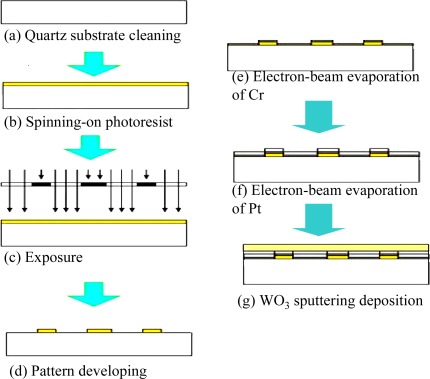
Overview of fabrication process for WO_3_ thin-film-based benzene sensor.

Figure 2 presents a schematic illustration of the fabrication process used to realize the current benzene sensor. Following the deposition of a thin layer of Cr (0.05 μm) which served as the adhesion layer for a Pt layer (0.2 μm) deposited using an electron-beam evaporation process on the quartz substrate, the WO_3_ sensing layer was sputtering deposited. A standard lift-off technique was employed to pattern the Pt micro-heater and IDEs. The resistance of the heater was designed to be 30 Ω. Following the WO_3_ sputtering deposition process, the sensor was annealed in dry air. In the annealing process, the sensor was heated from 25 °C to 500 °C over a period of 60 min, then maintained at a temperature of 500 °C for 90 min, and finally cooled back down to 25 °C over 2 h.

**Figure 3.**
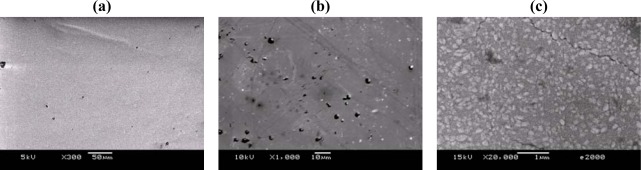
SEM images showing micrometer grain size of sputtered WO_3_ thin films as-sputtered condition with different deposition time: (a) 100 min, (b) 150 min and (c) 240 min.

**Figure 4.**
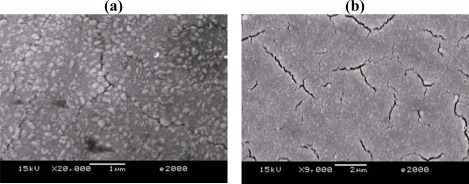
SEM images showing micrometer grain size of sputtered WO_3_ thin films with deposition time of 240 min at annealed condition: (a) X 20,000 and (b) X 9,000.

**Figure 5.**
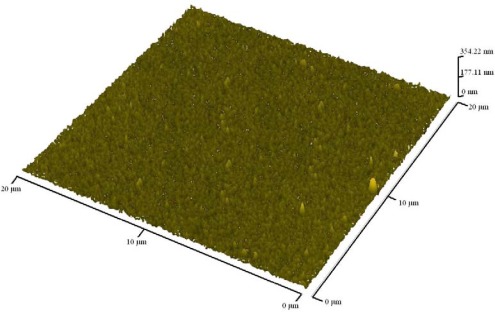
AFM image showing sub-micrometer grain size of sputtered WO_3_ thin films with deposition time of 240 min at annealed condition.

Figures 3 show SEM photographs of the as-deposited WO_3_ thin films. From inspection, voids were found on the substrate after a deposition time of 100 min but crystals of WO_3_ did not appear (Figure 3(a)). In Figure 3(b), WO_3_ crystals appeared with many voids after a deposition time of 150 min. After a deposition time of 240 min, WO_3_ crystals grew and connected with cracks which could enhance the sensitivity of the gas sensor due to the porosity on the sensing layer (Figure 3(c)). In Figure 3(c), the grain size is found to be approximately 0.2 μm, which is helpful to increase the contact area of the sensing layer with the sensed gas. Figures 4 present SEM images of sputtered WO3 thin film with deposition of 240 min after annealing. It can be seen smaller grains appeared after annealing. Hence, it is apparent that annealing enhances the sensing performance of the sensor by increasing the area/volume ratio in the sensing layer through reducing the grain size. Finally, the AFM image in Figure 5 reveals surface morphology of the sputtered WO_3_ thin film. It can be found the materials of the sputtering deposited thin film are uniform and the average roughness is 11.42 nm in the current study.

## 4. Results and Discussion

The diffraction pattern of the WO_3_ sensing layer was observed using an XRD measurement system (XRD-600 LabX, Shimadzu, Japan). The heating performance of the micro-heater was investigated using an IR thermometer (PT-3LF, OPTEX, Japan). The sensing performance of the device was characterized in a test chamber (30 cm × 30cm × 30 cm) by using an LCR meter (4230 LCR, Wayne Kerr Electronics, Taiwan) to record the signal response generated by changes in the benzene concentration over the range 0 to 20 ppm with a step-by-step concentration increase of 0.2 ppm.

### 4.1. Diffraction pattern of WO_3_ thin-film layer

**Figure 6.**
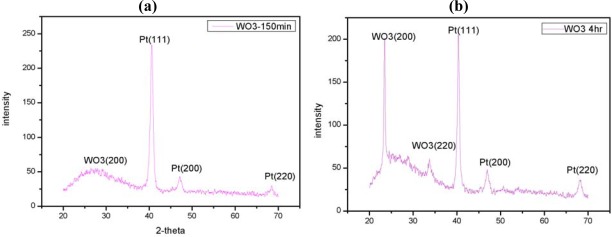
XRD diffraction patterns of WO_3_ film deposited at room temperature with different deposition time of: (a) 150 min and (b) 240 min. (Note: sputtering conditions as follows: Target, 99.99% WO_3_; RF power of target, 200 W; argon flow rate, 15 sccm; oxygen flow rate, 15 sccm; working pressure, 0.01 torr).

Figures 6 show the diffraction patterns of WO_3_ thin-film samples deposited at a sputtering temperature of 25 °C. In general, when a thin film is to be used as a sensing layer, it is desirable that the film has a non-random orientation. In Figure 6, the WO_3_ film was not formed with good crystalinity until that the specified sputtering annealing conditions resulted in a WO_3_ film with good crystallinity and a pronounced (002) preferred orientation with a deposition time of four hours.

### 4.2. Effect of applied power on micro-heater temperature

**Figure 7.**
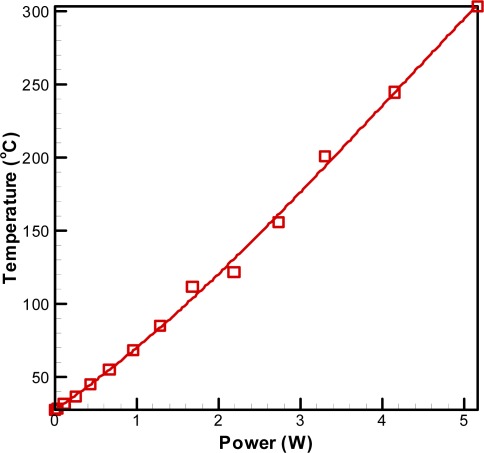
Variation of micro-heater temperature as function of applied power.

Figure 7 presents the variation of the micro-heater temperature with the applied power. It can be seen that the temperature increases linearly with increasing power at a rate of approximately 54°C/W.

### 4.3. Benzene concentration sensitivity

**Figure 8.**
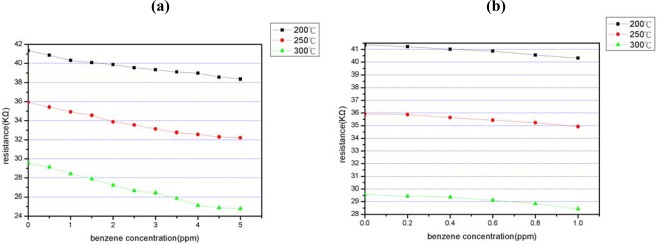
Sensitivity of benzene sensor as function of working temperature at benzene concentration of: (a) 1 – 5 ppm and (b) 0 – 1 ppm.

**Figure 9.**
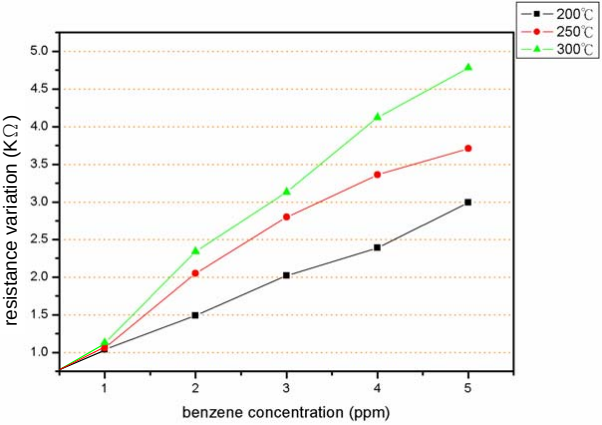
Effect of the sensor temperature on the benzene sensitivity.

As shown in Figures 8, linear dependency is observed between the resistance and the benzene concentration from 0 to 5 ppm in Figure 8(a) and from 0 to 1.0 ppm in Figure 8(b), respectively, at different sensor working temperatures (i.e., micro-heater temperatures). The slopes of the plotted lines represent the sensitivity of the device and are found to be 1.00 KΩ ppm^−1^ at 300 °C, 0.75 KΩ ppm^−1^ at 250 °C and 0.60 KΩ ppm^−1^ at 200 °C, respectively (Figure 9). Thus, it is apparent that the optimal working temperature of the benzene gas sensor is 300 °C. The minimum detection limit of the proposed sensor is also determined to be 0.2 ppm. Note that the detection limit is significantly lower than those reported for similar MEMS-based devices presented in previous studies.

### 4.4. Time response

**Figure 10.**
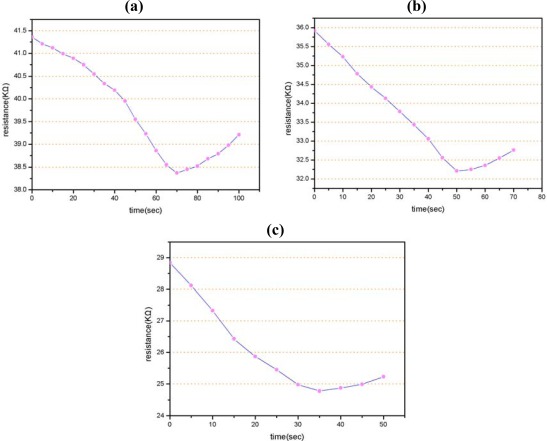
Time response of benzene sensor when benzene concentration is increased from 0 ppm to 6 ppm at working temperatures of: **(a)** 200 °C, **(b)** 250 °C, and **(c)** 300 °C.

In conventional gas measurement devices, the time required for benzene concentration measurement can vary from tens of seconds to minutes. However, a requirement exists for sensors with a real-time gas detection and measurement capability. Figures 10 present the time response of the benzene gas sensor at various working temperatures as the benzene concentration surrounding the sensor is increased from 0 to 6 ppm by abruptly removing a shutter near the sensor in the test chamber. From inspection, the average time constants of the gas sensor are found to be 70 s, 50 s and 35 s at working temperatures of 200 °C, 250 °C and 300 °C, respectively. Please note the resistance increases after the detection of the lowest resistance because the gas sensor reaches its saturation level.

### 4.5. Repeatability

**Figure 11.**
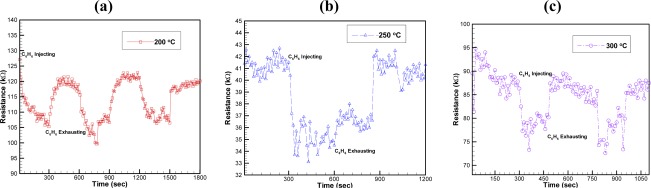
Repeatability of benzene sensor when benzene concentration is increased and decreased between 0 ppm and 20 ppm at working temperatures of: (a) 200 °C, (b) 250 °C, and (c) 300 °C.

Figures 11 present the repeatability results obtained by repeatedly increasing the concentration from 0 to 20 ppm, maintaining this concentration for a period, and then reducing to 0.

The results indicate that the benzene gas sensor has a recovery time of 120 s, 60 s and 30 s at working temperatures of 200 °C, 250 °C and 300 °C, respectively. The recovery time reduces as the sensing layer temperature increases to be 300 °C due to the offered thermal and kinetic energy for returning the chemical reactions within the sensing layer at the temperature for recovery characteristics.

## 4. Conclusions

This study has demonstrated a novel MEMS-based benzene gas sensor featuring a thin WO_3_ sensing layer. Pt micro-heater/IDE are deposited on a quartz substrate and the WO_3_ film is then deposited directly on the micro-heater/IDE. The integrated micro-heater simplifies the detection setup and provides the advantages of good temperature control and low power consumption. The proposed sensor has a high degree of sensitivity, a low detection limit, a rapid response time, and a quick recovery time. The sensor is suitable not only for industrial process monitoring, but also for the detection of benzene concentrations in indoor environments in order to safeguard human health.
